# Association Between Geographic Elevation, Bone Status, and Exercise Habits: The Shimane CoHRE Study

**DOI:** 10.3390/ijerph120707392

**Published:** 2015-06-30

**Authors:** Miwako Takeda, Tsuyoshi Hamano, Kunie Kohno, Shozo Yano, Kuninori Shiwaku, Toru Nabika

**Affiliations:** 1 Center for Community-Based Health Research and Education (COHRE), Organization for the Promotion of Project Research, Shimane University, 223-8 Enya-cho, Izumo, Shimane 693-8501, Japan; E-Mails: thamano@med.shimane-u.ac.jp (T.H.); nine_kk@med.shimane-u.ac.jp (K.K.); syano@med.shimane-u.ac.jp (S.Y.); shiwaku@jn.shimane-u.ac.jp (K.S.); nabika@med.shimane-u.ac.jp (T.N.); 2 Department of Laboratory Medicine, Shimane University School of Medicine, 89-1 Enya-cho, Izumo, Shimane 693-8501, Japan; 3 Department of Functional Pathology, Shimane University School of Medicine, 89-1 Enya-cho, Izumo, Shimane 693-8501, Japan

**Keywords:** bone status, quantitative ultrasound, geographic elevation, aging population

## Abstract

In recent years, there has been growing interest in the association between the residential environment and health. The association between residential environment (*i.e.*, geographic elevation) and bone status is unknown. Furthermore, these associations could differ by exercise habits due to the chronically greater daily activity caused by steep slopes in mountainous areas. The aim of this study was to test whether the association between bone status of elderly people measured using quantitative ultrasound (QUS) and elevation varied according to the exercise habits in a mountainous area population. Data were collected from a cross-sectional study conducted during 2012–2013. QUS value was expressed as a proportion of the young adult mean (%YAM), with higher scores donating better bone status. After excluding subjects with missing data, we analyzed the data for 321 men and 500 women. Our results indicate that %YAM was not associated with elevation among men, or among women with exercise habits. However, elevation was associated with %YAM among women without exercise habits. Our results highlight the importance of considering residential environment and exercise habits when establishing promotion strategies to maintain bone status of the elderly people who live in rural mountainous areas.

## 1. Introduction

In recent years, osteoporosis has become an important public health concern, and the estimated number of osteoporotic patients aged 40 years or over in Japan is estimated at 12.8 million (3 million men and 9.8 million women) [1]. The bone status test is performed in individuals who are at-risk for osteoporosis. For example, a lower bone mineral density (BMD) is known to increase the risk of fractures, which reduce quality of life among elderly people [1,2,3]. Decreasing BMD is also a characteristic among menopausal women [4]. Therefore, it is important to minimize the decrease in the maximum BMD that occurs in the aging population.

Several risk factors for declining bone status have been postulated, including sociodemographic factors (e.g., age and sex), lifestyle and diet (e.g., physical activity, smoking, drinking, and calcium intake), and health conditions and genetic factors (e.g., overweight or parental hip fracture) [1,2,3,4,5,6,7,8,9]. In addition, the residential environment (*i.e.*, geographic elevation) would be a potential risk factor in rural mountainous areas [10,11,12]. In a previous study, it has been indicated that obese people who live at high elevations were more likely to have chronic knee pain compared to those who live at low elevations [10]. This association is likely because walking up and down steep slopes may increase the mechanical load on the knees. 

In Japan, approximately 70% of the landmass is covered by hilly and mountainous areas that extend from the outer plains to the mountains [13]. It is hypothesized that elderly people who live at high elevations have a better bone status compared to those who live at low elevations due to the chronically greater daily activity caused by steep slopes in rural mountainous areas. Furthermore, these associations could differ according to exercise habits. To the best of our knowledge, no previous studies have been conducted to test this hypothesis. The aim of this study was to test whether the association between bone status, measured using quantitative ultrasound (expressed as a proportion of the young adult mean, with higher scores donating better bone status; %YAM), and elevation varied according to the exercise habits in an elderly population of mountainous area.

## 2. Methods

### 2.1. Study Population

Data were collected from a cross-sectional study conducted during 2012–2013. This study was part of the Shimane Community-based Health Research and Education (CoHRE) Study, which was designed to examine the determinants of various lifestyle-related diseases, including bone status. The Shimane CoHRE study was conducted by Shimane University in Japan, and was undertaken in collaboration with a health examination program that was conducted in Unnan city. This city is located in a rural mountainous area in the southern part of Shimane prefecture, Japan. People who were not ADL-independent were excluded, and there were no monetary or other incentives to participate this study.

The residents who live in this city have two options for receiving their regular health examinations. The first option is a group examination that is conducted at public health centers, and the second option is an individual examination that is conducted at medical institutions. We were permitted to use and analyze group examination data for this study. After excluding subjects who had missing data, we analyzed the data for 321 men and 500 women. The study protocol was approved by the ethics committee of Shimane University School of Medicine (2010), and written informed consent was obtained from all participants.

### 2.2. Bone Measurement

Bone status was measured using quantitative ultrasound (QUS) (Benus α; Ishikawa Seisakusho, Ltd., Ishikawa, Japan). QUS has some advantages, such as no exposure to radiation, low cost, and portability [14,15]. QUS enables the evaluation of bone quality, especially the microarchitecture at the calcaneus. The estimated value which compared to young adult mean (%YAM) with the same gender of examine, *i.e.*, 100% means same value as healthy young men or women.

### 2.3. Elevation

Geographic information systems (ArcGIS software, version 10.0; Environmental Systems Research Institute, Redlands, CA, USA) was employed for database queries and used to estimate elevation based on the individual’s address. The elevation for each participant was assessed using the ArcGIS ready-to-use dataset of digital elevation models. Log-transformed elevation was used in the analysis.

### 2.4. Other Measures

We also considered the following variables in the analysis: age (years, analyzed as a continuous variable), sex (men *vs.* women), body mass index (BMI in kg/m^2^, analyzed as a continuous variable), parental hip fracture(yes *vs.* no), current smoking (yes *vs.* no), drinking alcohol (yes *vs.* no), car driver (yes *vs.* no), exercise habits (>30 min/day of moderate–vigorous exercise, more than twice per week for at least 1 year; yes *vs.* no), calcium intake (more than the Japanese age-adjusted standard value, yes *vs.* no) [16,17], and age at menopause (years, analyzed as a continuous variable). Brief self-administered DHQ (BDHQ) that asks about the consumption frequency of selected foods was used to calculate calcium intake [18].

### 2.5. Statistical Analysis

Descriptive statistics were calculated for all characteristics, and the χ2 and Mann-Whitney U tests were used to compare the characteristics according to the participants’ exercise habits. Multivariable linear regression models were developed to calculate the regression coefficient, standard error, and p-value. Bone status (%YAM) was used as an independent variable, and elevation (log10-transformed), age, BMI, age at menopause (women only), parental hip fracture, current smoking (men only), drinking alcohol, car driver, and calcium intake were used as dependent variables. *p*-values of <0.05 were considered statistically significant, and all statistical analyses were performed using IBM SPSS Statistics 20 (IBM Corporation, Tokyo, Japan).

## 3. Results

The characteristics of the study participants are shown in Table 1. There were no statistically significant differences in the bone status, age, BMI, parental hip fracture, current smoking, drinking alcohol, and car driver of men who did and did not have an exercise habits. However, elevation and calcium intake were significantly different between the men who did and did not have an exercise habits. Among the women, there were no statistically significant differences in bone status, elevation, BMI, age at menopause, parental hip fracture, current smoking, drinking alcohol, and car driver. However, age and calcium intake were significantly different between the women who did and did not have an exercise habits. 

**Table 1:** Characteristics of the study participants.

	Men						Women				
	Exercise habits						Exercise habits				
	Yes (n = 115)		No (n = 206)				Yes (n = 187)		No (n = 313)		
	n	% ormean (SD)	n	% ormean (SD)	*p*		n	% ormean (SD)	n	% ormean (SD)	*p*
Bone status (%YAM), %	115	90.0 (10.8)	206	90.4 (11.7)	0.876		187	83.4 (8.9)	313	83.2 (10.6)	0.408
Elevation, m	115	71.2 (60.2)	206	124.6 (107.6)	<0.001		187	112.4 (115.4)	313	106.3 (97.0)	0.488
Age, years	115	71.6 (7.2)	206	70.9 (7.7)	0.247		187	70.3 (6.1)	313	69.3 (7.0)	0.022
Body mass index, kg/m^2^	115	22.4 (2.7)	206	22.4 (3.2)	0.969		187	22.0 (2.8)	313	21.8 (3.0)	0.453
Age at menopause, years							187	49.8 (4.7)	313	49.2 (4.7)	0.118
Parental hip fracture, %	9	7.8	22	10.7	0.407		21	11.2	27	8.6	0.339
Current smoking, %	16	13.9	25	12.1	0.647		1	0.5	1	0.3	0.712
Drinking alcohol, %	84	73.0	161	78.2	0.302		54	28.9	81	25.9	0.465
Car driver, %	112	97.4	202	98.1	0.695		114	61.0	212	67.7	0.124
Calcium intake, %	70	60.9	100	48.5	0.034		127	67.9	181	57.8	0.025

n, number of participants; SD, standard deviation; %YAM, proportion of the young adult mean.

Table 2 shows the results of the multivariable linear regression analysis according to exercise habits among men. For the group with exercise habits, elevation was not significantly associated with %YAM (regression coefficient = 6.545, *p* = 0.123), although parental hip fracture was significantly associated with %YAM (regression coefficient = 8.671, *p* = 0.031). For the group without exercise habits, elevation was not significantly associated with %YAM (regression coefficient = –0.691, *p* = 0.780), although age was significantly associated with %YAM (regression coefficient = –0.322, *p* = 0.006).

**Table 2:** Multivariable linear regression analysis among men.

	Exercise habits (n = 115)					No exercise habits (n = 206)			
	B	SE	t	*p*-value		B	SE	t	*p*-value
Elevation	6.545	4.207	1.556	0.123		–0.691	2.475	–0.279	0.780
Age, years	–0.120	0.165	–0.727	0.469		–0.322	0.115	–2.805	0.006
Body mass index, kg/m^2^	0.143	0.406	0.352	0.725		0.085	0.260	0.326	0.745
Parental hip fracture, no *vs.* yes	8.671	3.957	2.191	0.031		1.911	2.644	0.723	0.471
Current smoking, no *vs.* yes	–2.983	3.268	–0.913	0.363		1.830	2.594	0.705	0.481
Drinking alcohol, no *vs.* yes	0.490	2.341	0.210	0.834		0.639	1.995	0.320	0.749
Car driver, no *vs.* yes	1.406	6.536	0.215	0.830		–0.393	6.010	–0.065	0.948
Calcium intake, no *vs.* yes	3.011	2.130	1.414	0.160		1.019	1.644	0.619	0.536

SE: standard error. Independent variable: bone status measured using QUS. Dependent variables: elevation (log10-transformed), age, body mass index, parental hip fracture, current smoking, drinking alcohol, car driver, and calcium intake.

Table 3 shows the results of the multivariable linear regression analysis according to exercise habits among women. For the group with exercise habits, elevation was not significantly associated with %YAM (regression coefficient = 2.298, *p* = 0.216), and none of the other factors were significantly associated with %YAM. For the group without exercise habits, elevation (regression coefficient = 3.686, *p* = 0.032), age (regression coefficient = –0.363, *p* < 0.001), and BMI (regression coefficient = 0.946, *p* < 0.001) were significantly associated with %YAM.

**Table 3:** Multivariable linear regression analysis among women.

	Exercise habits (n = 187)					No exercise habits (n = 313)			
	B	SE	t	*p*-value		B	SE	t	*p*-value
Elevation	2.298	1.852	1.241	0.216		3.686	1.710	2.155	0.032
Age, years	–0.192	0.118	–1.630	0.105		–0.363	0.089	–4.099	<0.001
Body mass index, kg/m^2^	0.177	0.227	0.778	0.437		0.946	0.184	5.144	<0.001
Age at menopause, years	0.189	0.140	1.350	0.179		0.056	0.117	0.480	0.632
Parental hip fracture, no *vs.* yes	1.089	2.047	0.532	0.595		1.479	1.984	0.745	0.457
Drinking alcohol, no *vs.* yes	1.955	1.481	1.320	0.188		1.047	1.285	0.815	0.416
Car driver, no *vs.* yes	0.968	1.476	0.656	0.513		0.973	1.350	0.721	0.472
Calcium intake, no *vs.* yes	–0.096	1.402	–0.068	0.946		2.081	1.121	1.856	0.064

SE: standard error. Independent variable: bone status measured using QUS. Dependent variables: elevation (log10-transformed), age, body mass index, age at menopause, parental hip fracture, drinking alcohol, car driver, and calcium intake.

## 4. Discussion

Our results advance the existing debate regarding the associations between bone status and residential environment. For example, previous studies have determined the presence of geographical differences in bone status, indicating that bone status might be affected by environmental factors [3,19]. In addition, a previous study has found that daily activity is associated with a lower rate of hip fracture [5]. These evidences can be used to support our finding that elevation and exercise habits were associated with bone status. Further studies should be conducted to examine whether the amount of daily activities is greater at higher elevations compared to lower elevations.

Previous studies have found sex-related differences in the risk of osteoporosis [1,2,3]. Our results also confirmed that the average bone status in women (83.3 ± 11.4%) was statistically lower than that in men (90.3 ± 9.9%). This difference may be used to explain why the associations between bone status and residential environment only varied among women according to exercise habits. Healthcare professionals should consider these factors when formulating efficient health plans to maintain bone status, especially for women who live in rural mountainous areas.

The present study has two major strengths. First, to the best of our knowledge, this was the first study in which the association between elevation and bone status according to exercise habits was determined. Second, bone status was measured using a validated objective method that is considered more accurate than subjective methods (*i.e.*, questionnaire surveys). This study also has several potential limitations. First, the data is not an equitably representative sample. A selection bias caused by non-respondents was present and may have influenced the associations as shown in our results. Second, the findings may be mediated by factors that were not assessed in this study. Third, dual-energy X-ray absorptiometry is more commonly used to evaluate bone status. However, QUS method is no radiation exposure and low cost, so it could improve utility in the community setting [14]. In addition, QUS could be useful for the primary prevention in the community due to portability. Finally, we could not establish the temporal order of causality because the present study used a cross-sectional design.

## 5. Conclusions

Our results indicate that specific associations between elevation and bone status, measured using QUS, are observed among the elderly women without exercise habits. Our results highlight the importance of considering residential environment and exercise habits when establishing promotion strategies to maintain bone status among persons who live in rural mountainous areas. Longitudinal research is required to confirm these findings, and to explore their potential mechanisms.
